# Recent Progress in the Conversion of Methylfuran into Value-Added Chemicals and Fuels

**DOI:** 10.3390/molecules29132976

**Published:** 2024-06-22

**Authors:** Wei Wang, Jiamin Yan, Mengze Sun, Xiufeng Li, Yanqing Li, Ling An, Chi Qian, Xing Zhang, Xianzhao Shao, Yanping Duan, Guangyi Li

**Affiliations:** 1 Shaanxi Key Laboratory of Catalysis, School of Chemistry and Environment Science, Shaanxi University of Technology, Hanzhong 723001, China; 17829847382@163.com (J.Y.); 17809135535@163.com (M.S.); lyq1690109355@163.com (Y.L.); anling202425@163.com (L.A.); qianchi0720@163.com (C.Q.); xianzhaoshao@snut.edu.cn (X.S.); duanyanping2006061@126.com (Y.D.); 2 Hanzhong Institute of Agricultural Science, Hanzhong 723000, China; lixiufengmm@126.com; 3 State Key Laboratory of Catalysis, Dalian Institute of Chemical Physics, Chinese Academy of Sciences, Dalian 116023, China; zjipuyu@163.com

**Keywords:** 2-methylfuran, hydroxyalkylation/alkylation, Diels–Alder, acylation reaction, fuel

## Abstract

2-methylfuran is a significant organic chemical raw material which can be produced by hydrolysis, dehydration, and selective hydrogenation of biomass hemicellulose. 2-methylfuran can be converted into value-added chemicals and liquid fuels. This article reviews the latest progress in the synthesis of liquid fuel precursors through hydroxyalkylation/alkylation reactions of 2-methylfuran and biomass-derived carbonyl compounds in recent years. 2-methylfuran reacts with olefins through Diels–Alder reactions to produce chemicals, and 2-methylfuran reacts with anhydrides (or carboxylic acids) to produce acylated products. In the future application of 2-methylfuran, developing high value-added chemicals and high-density liquid fuels are two good research directions.

## 1. Introduction

The sustenance and advancement of human societies are fundamentally dependent on energy as a crucial material resource. Progress in energy development, coupled with its efficient utilization and per capita consumption, serves as a critical metric for assessing technological advancements, the quality of life, and the degree of societal progress. The environment is a variety of natural factors that can directly or indirectly influence human survival and development. The rapid consumption of energy has brought about environmental problems. Energy and environment have become one of the current hot topics of concern [1,2,3,4].

**Figure 1:**
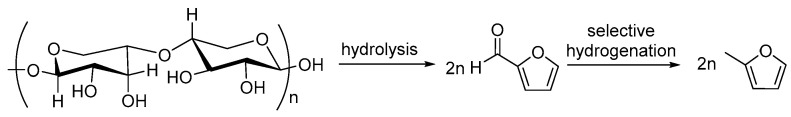
The pathway for converting hemicellulose to 2-methylfuran.

In the context of the “dual carbon goal”, lignocellulosic biomass with an annual productivity of over 180 billion tons has been identified as a promising raw material in the search for alternative resources to produce low-carbon future commodity chemicals and transportation fuels [5,6]. Lignocellulose includes lignin, cellulose, and hemicellulose [7,8,9], which has the potential to be transformed into chemicals and fuels through catalytic conversion [10,11]. 2-methylfuran is an organic compound with the chemical formula C_5_H_6_O. 2-methylfuran is used as a chemical raw material to prepare acetylpropanol, pentadiene, pentanediol, etc. It can also be used in the pharmaceutical field to synthesize vitamin B1 (anti-neuroinflammatory drug), chloroquine phosphate, and primaquine phosphate (anti-dysentery drug). It is also a good solvent. It is an important organic intermediate with important applications in the fields of chemical, pharmaceutical, pesticide, and energy. Meanwhile, 2-methylfuran has a higher octane number and higher energy density than ethanol, making it a biofuel alternative to gasoline [12,13]. The synthesis of 2-methylfuran involves a selective hydrogenation step of furfural [14] derived from the hydrolysis and dehydration processes of hemicellulose [15,16,17,18]. The pathway for converting hemicellulose to 2-methylfuran is shown in Figure 1.

Selective hydrogenation of furfural to 2-methylfuran was achieved on Ni-Fe bimetallic catalyst supported by silica, and good results were obtained. The emergence of Ni-Fe bimetallic alloy phase favors the production of 2-methylfuran [15]. A bimetallic Ni-Cu alloy catalyst loaded on ZSM-5 zeolite was carried out using the impregnation method, it was employed in the hydrodeoxygenation of furfural to prepare 2-MF as well. Adding a suitable quantity of Ni in Cu/ZSM-5 increased the conversion rate of furfural, and the adsorption of furfural and H_2_ was enhanced. Using the optimized 2Ni-6Cu/ZSM-5 catalyst that contains 2 wt% Ni and 6 wt% Cu on ZSM-5, the reaction was reacted at 220 °C for 30 min, resulting in a yield of 78.8 wt% of 2-MF [16]. Using the Ru/NiFe_2_O_4_ catalyst, research was conducted on the hydrogenation of furfural to 2-methylfuran, with 2-propanol serving as the source of hydrogen. Under the specified mild conditions (2.1 MPa N_2_ and 180 °C), the conversion rate of furfural is above 97% and the yield of 2-methylfuran is as high as 83% [18]. The pathway of furfural conversion from hemicellulose to 2-methylfuran at the molecular level was elucidated using isopropanol as the hydrogen source on a Ru/RuOx/C bifunctional catalyst by combining isotopic labeling with kinetic studies. Furfural carbonyl is hydrogenated to furfuryl alcohol through a process that involves intermolecular hydride transfer mediated by Lewis acid, and the hydrogenation of furfuryl alcohol is chiefly carried out through the activation of the ring by metal and Lewis acid sites [19]. On the Mg/Fe/O catalyst, methanol serves as the hydrogen source, and when the reaction occurs between 300 and 400 °C, it mainly forms 2-methylfuran. Under these reaction conditions, using Mg/Fe/O, the quantitative conversion of furfural was observed with a 2-methylfuran yield of 83% [20]. The wet impregnation method was employed to produce single metal and bimetallic catalysts on activated carbon with varying amounts of Pt and Co. Under PtCo/C bimetallic catalyst, a 2-methylfuran yield of 59% was obtained at 0.5 MPa H_2_ pressure and 180 °C [21]. Multiple types of iridium catalysts supported by carbon have been prepared to facilitate the direct hydrogenation of furfural into 2-methylfuran. Among them, under H_2_ pressure of 100 psig, 5% Ir/C shows outstanding properties—total conversion of furfural and 2-methylfuran selectivity up to 95%. Both the metal (Ir) and oxide (IrO_2_) forms of Ir catalyze the initial stage of the hydrogenation reaction, including furfural to furfuryl alcohol, followed by further hydrogenation, resulting in the production of 2-methylfuran [22]. The reaction of furfural-catalyzed hydrogenation transfer to prepare 2-methylfuran was studied on a CuZnAl catalyst using isopropanol as the hydrogen donor, indicating that furfural is converted to 2-methylfuran through furfuryl alcohol. The cracking of the C-O (H) bond, identified as the rate-determining step, has a positive correlation with the copper content. H_2_ provides reduced surface Cu^0^ and Cu^+^ species for the pre-reduction of CuZnAl. On the Cu2.5Zn-Al-600 catalyst, a 2-methylfuran yield reached 72% under atmospheric pressure at 180 °C in N_2_ [23]. On bimetallic NiCuAl and CoCuAl catalysts, 2-propanol was used as the hydrogen source to systematically study the conversion of furfural to 2-methylfuran and furfuryl alcohol. The influencing factors of reaction time and temperature were investigated and optimized, and NiCuAl and CoCuAl catalysts achieved the highest yields and selectivity of the two main products. The excellent performance of bimetallic catalysts is attributed to the stronger Lewis acidic centers on the catalyst surface [24]. An extremely efficient nickel cobalt bimetallic catalyst was synthesized through the coprecipitation method for the catalytic hydrogenation of furfural, a biomass platform product, aiming to produce high value-added chemical 2-methylfuran. On the Ni_10_Co_5_-MgAlO catalyst, when the Ni/Co molar ratio is 2 and 2-butanol is used as the solvent, the conversion rate of furfural is 100%, and the selectivity for 2-MF is 92.3% [25]. Low-cost CuFe catalysts were synthesized using citric acid complexation method. Using isopropanol as a hydrogen source, the catalyst, activated in situ after calcination was used for the catalytic performance of furfural conversion to 2-methylfuran and furfuryl alcohol. Through joint observation of XRD and non-in-situ XANES, metal Cu with a small amount of Cu_2_O and magnetite Fe_3_O_4_ was generated on the catalyst after the conversion of furfural to 2-methylfuran and furfuryl alcohol, as the active substance of the reaction [26]. The highly dispersed MoP catalyst supported with SiO_2_ was created using the impregnation method, with citric acid serving as an adjuvant. The prepared MoP/SiO_2_ catalyst exhibits excellent performance in converting furfural to 2-methylfuran through selective hydrogendeoxygenation. Within a continuous fixed-bed reactor under moderate operating conditions (1.0 MPa, 120 °C, WHSV: 0.3 h^−1^) and using over 20% MoP/SiO_2_, furfural was completely converted, and the selectivity for 2-methylfuran was 96.3%. The surface acidity of MoP and the oxygen affinity of Mo improve the adsorption of furfural and facilitate the cleavage of the C-O bond in intermediate furfuran alcohols, making 2-methylfuran have high selectivity [27]. A 20 wt% Co CoOx/AC catalyst was prepared, with an adjustable ratio of metal to oxide. Under optimized reaction conditions (120 °C, 2.5 MPa H_2_, 5 h), the yield of 2-methylfuran from furfural reached 87.4%, and the catalyst performance did not significantly decrease after five cycles. The combination of physical and chemical characterization, catalytic activity data, and DFT calculation results indicates that the active components in the catalyst exist in the form of Co^0^ species and CoOx species, explaining the reaction mechanism [28].

A hollow nitrogen-doped carbon cage bimetallic catalyst (CuCo/NC) containing CuCo alloy was prepared using ZIF-67 as an auxiliary template. The catalyst exhibited excellent catalytic performance, with complete conversion of furfural and a yield of 95.7% for 2-methylfuran. Physical and chemical characterization and density functional theory calculations indicate that the introduced Cu species regulate the activity and selectivity of the catalyst through two aspects. On the one hand, Cu species disrupt the electronic structure of Co, causing the adsorption conformation of furfural on the catalyst surface to change from flat to vertical, successfully hindering the hydrogenation of the furan ring and improving the selectivity of 2-methylfuran. On the other hand, the CuCo (111) site promotes hydrogen dissociation and C-O bond cleavage, thereby promoting the formation of 2-methylfuran [29].

Using ReOx and WOx modified Cu/Al_2_O_3_ catalysts, furfural (FAL) was selectively hydrogenated into 2-methylfuran (2-MF) and 2-methyltetrahydrofuran (2-MTHF) in a fluidized bed reactor. Through catalyst characterization analysis, it was found that the metal support interaction was enhanced on Cu/Al_2_O_3_ catalysts modified with ReOx and WOx, while also affecting the acid strength and quantity. Operating at 220 °C, the ReCuAl catalyst achieved a combined yield of 2-MF and 2-MTHF at 80.4%, the WCuAl catalyst reached 89.0%, and the CuAl catalyst achieved a yield of 54.0% [30]. A route has been developed for the tandem conversion of xylose into 2-methylfuran. Firstly, xylose is converted to furfural on Hβ zeolite, and then furfural is catalyzed to 2-methylfuran on 0.5NiCu/C. Compared to the two-step reaction, the application of the xylose cascade strategy increased the yield of 2-methylfuran by 41.5% [31]. The catalytic conversion of furfural to 2-methylfuran was systematically studied on a series of CuZn/FeOx catalysts derived from sol–gel. The characterization study shows that there are Cu^0^ and Cu^+^ species on the surface of sol–gel-derived samples, which is mainly due to the co reduction of homogeneous ternary compositions. On CuZn/FeOx (280), the final yield of 2-methylfuran reached 91% at 210 °C [32].

In this work, it is summarized that in recent years, 2-methylfuran and biomass-derived carbonyl compounds have undergone hydroxyalkylation/alkylation, followed by Diels–Alder cycloaddition reactions with olefins and acylation reactions with anhydrides or carboxylic acids to generate biomass liquid fuels or high-value-added chemicals.

## 2. Hydroxyalkylation/Alkylation of Methylfuran

**Figure 2:**
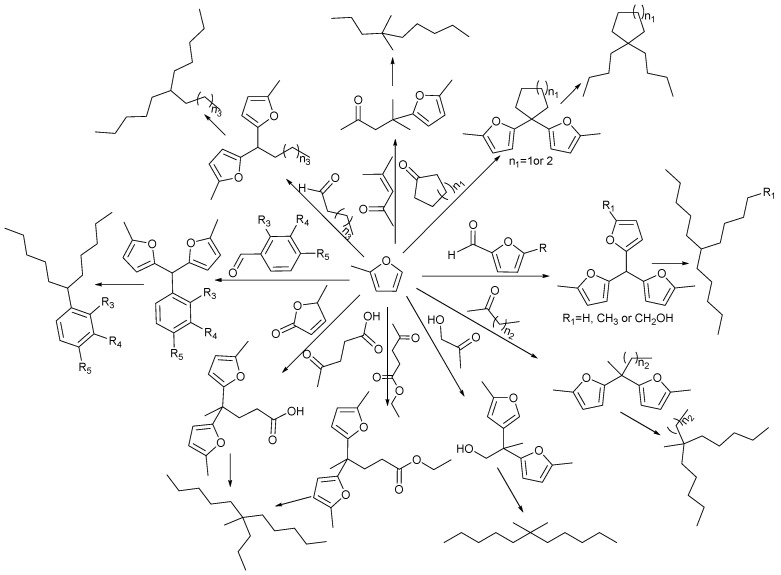
Pathway for the synthesis of liquid fuels from methylfuran and biomass-derived aldehydes, ketones, esters, etc.

Methylfuran undergoes hydroxyalkylation/alkylation reactions with aldehydes, ketones, esters, etc., derived from biomass to produce long carbon chain oxygen-containing compounds. These compounds can be hydrodeoxygenated to obtain liquid fuels with corresponding carbon numbers. The reaction pathway is shown in Figure 2.

The hydroxyalkylation/alkylation reaction of 2-methylfuran with butanal, 5-hydroxymethylfurfural, and 5-methylfurfural to produce fuel precursors, which then are hydrodeoxygenated to produce alkanes with corresponding carbon chain lengths. The catalysts involved in the hydroxyalkylation/alkylation reaction process include p-toluenesulfonic acid, sulfuric acid, and Amberlyst-15. The hydroxyalkylation/alkylation products were hydrodeoxygenated on platinum carbon and platinum alumina catalysts, resulting in a liquid alkane yield of 96.5% [33]. 2-methylfuran and 5-methylfurfural undergo hydroxyalkylation/alkylation reaction under the catalysis of sulfuric acid or p-toluenesulfonic acid, and the resulting product is hydrodeoxygenated on platinum supported carbon, and platinum supported titanium oxide. The yield of C_9_-C_16_ alkane liquid fuel is 92.1% [34]. The hydroxyalkylation/alkylation reaction results of 5-methylfurfural and 2-methylfuran are shown in Table 1.

The hydroxyalkylation/alkylation of butanal with 2-methylfuran can also be carried out on Beta (comm), Beta (nano), Beta (OH^−^), Beta (F^−^), MCM-41 (Si/Al ratio = 15), MCM-41 (Si/Al ratio = 28), ITQ-2, and Dowex 50WX2-100 catalysts. On these catalysts, with 2-methylfuran and butanal mixed in a 2:1 molar ratio, the catalyst was added, followed by stirring the reaction mixture mechanically and heating it to 50 °C for 8 h. The range of yields for 2,2′-butylidenebis [5-methylfuran] is distributed between 16% and 86%. The catalytic performance of ITQ-2 is the best, with the yield of 2,2′-butylidenebis [5-methyluran] reaching 86% [35]. The hydroxyalkylation/alkylation reaction results of butanal and 2-methylfuran are shown in Table 2.

**Figure 3:**
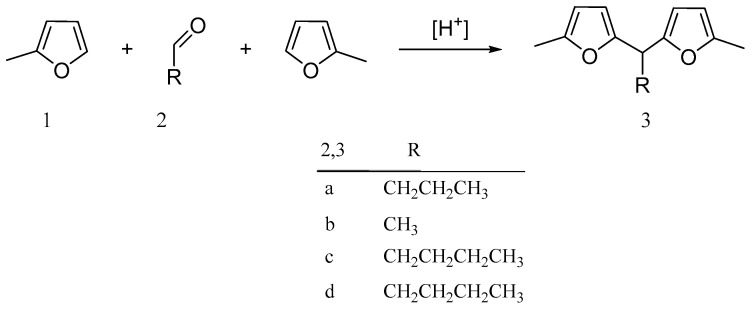
Hydroxyalkylation/alkylation of 2-MF (**1**) with butanal (**2a**), ethanal (**2b**), propanal (**2c**), and pentanal (**2d**) [35].

The reaction routes of hydrolkylation/alkylation of Sylvan (**1**) with butanal (**2a**), ethanal (**2b**), protonal (**2c**), and pentanal (**2d**) are shown in Figure 3, and the reaction conditions and results are shown in Table 3.

The hydroxyalkylation/alkylation reactions of 2-methylfuran (2-MF) with acetone and butanal were studied over an array of solid acid catalysts (Nafion212, 72% H_2_SO_4_, Amberlyst-15, Amberlyst-36, Nafion115, MC-SO_3_H, CMK-3-SO_3_H, ZrP, AC-SO_3_H, H-ZSM-5, and H-Y). In the study of various catalysts, Nafion-212 showed superior activity and stability. Under optimized conditions (323 K, 48 h), the yield of the hydroxyalkylation/alkylation reaction product between acetone and methylfuran is 72.4%. The yield of the alkylation/alkylation reaction product between methylfuran and butanal is 89.5% under 323 K, 2 h. Surface butanal has higher activity and selectivity than acetone [36].

Through hydrothermal treatment with a sodium hydroxide (NaOH) solution and subsequent ion exchange with an acidic solution, TiO_2_ P25 was transformed into proton titanate nanotubes (PTNTs), significantly increasing the specific BET surface area and acidity of the catalyst (including the amount and strength of acid sites) and causing the generation of Brønsted acid sites. PTNT is used for hydroxyalkylation/alkylation (HAA) of 2-methylfuran (2-MF) derived from lignocellulose and butanal. When contrasted with alternative inorganic solid acids, PTNT exhibits superior catalytic performance and selectivity. Under optimized conditions, the yield of 5,5′-(butane-1,1-diyl)bis(2-methylfuran) can reach 77%. PTNT indeed proves to be viable for the HAA (or alkylation with isopropylacetone) reaction between furfural and 2-MF and acetone as well [37].

The main reaction between methylfuran and isopropylidene acetone is a 1:1 addition reaction, Table 4 presents the reaction conditions and the corresponding experimental results. The yield of 4-methyl-4-(5-methyluran-2-yl) pentan-2-one can reach 89% [35].

The process of alkylating mesityl oxide with 2-methylfuran was executed using various solid acid catalysts. Among the catalysts studied, Nafion-212 resin demonstrates the greatest catalytic efficiency and reliable stability, which is related to its higher acid strength. When the conditions are optimized, the yield of the product obtained from the alkylation of 2-methylfuran with mesityl oxide can reach 70% [38]. The alkylation reaction results of 2-methylfuran with mesityl oxide are shown in Table 4.

The alkylation products of mesityl oxide and 2-methylfuran are hydrolyzed to form (or a one-pot reaction of trimethylene oxide, 2-methylfuran, and water) triketones. The solvent-free intramolecular aldol condensation reaction of triketones is followed by h Hydrodeoxygenated to obtain high-density (0.82 g mL^−1^) and low-freezing-point (217–219 K) jet-fuel-branched cyclic alkanes [39].

**Figure 4:**
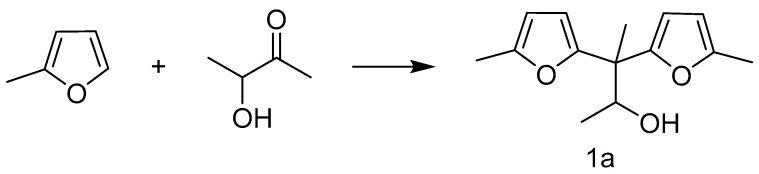
The comparison of the reactivity of acetoin with 2-MF in the HAA. Reaction conditions: 2-MF (22 mmol), acetoin (10 mmol), TFA-ZrO_2_ (0.1 g), 333 K, 2 h [40].

In the catalytic alkylation/alkylation reaction of 2-methylfuran with acetoin, acetone, and butyraldehyde on a zirconia-supported trifluoromethanesulfonic acid catalyst (TFA-ZrO_2_), fuel precursor **1a** (93%) was obtained with high yields [40]. The reaction pathway is shown in Figure 4.

Functionalization of the pore pores of zeolite β was carried out using poly (phenylethylsulfonic acid) (PE Betas), and the acidity, hydrophobicity, and confinement were integrated into the solid material. In the hydroxyalkylation/alkylation of 2-methylfuran with acetone, these PE Betas, conventional H-Betas, mesoporous polystyrene ethyl sulfonic acid functionalized silica gel (PE-SiO_2_), and commercial sulfonic acid catalysts (p-toluene sulfonic acid, Nafion NR50) were evaluated. PE-Betas exhibit excellent turnover number (TON) due to the high acidic site strength and hydrophobicity generated by poly (ethylsulfonic acid) [41].

A variety of solid acid catalysts (Nafion212, Nafion1135, Nafion115, Amberlyst-15, Amberlyst-36, MC-SO_3_H, AC-SO_3_H, ZrP, CMK-3-SO_3_H, and SO_4_^2−^/Al_2_O_3_) were used for the HAA reaction of methylfuran derived from lignocellulose with furfural, ethyl levulinate, and acetone. In comparison to the other catalysts studied, Nafion-212 resin exhibited the highest activity and stability. Under optimized conditions (2 h, 323 K), the HAA product yield of furfural and 2-MF can reach 75%. Under these reaction conditions, methylfuran undergoes hydroxyalkylation/alkylation with ethyl levulinate and acetone to produce ethyl 4,4-bis(5-methylfuran-2-yl) pentanoate and 5,5′-(propane-2-diyl)bis(2-methylfuran) in yields of 24% and 14%, respectively [42].

N-methyl (or butyl), N-butyl sulfonic acid bisulfate imidazole ionic liquids were synthesized using N-methyl (or butyl) imidazole. N-methyl (or butyl) imidazole was used to synthesize N-methyl (or butyl), N-butyl sulfonic acid trifluoromethyl sulfate imidazole ionic liquids, and they were used to catalyze hydrogenation/alkylation reactions of 2-methylfuran with furfural. N-butyl, N-butyl sulfonic acid bisulfate imidazole ionic liquids and N-butyl, N-butyl sulfonic acid trifluoromethyl sulfonate imidazole ionic liquids exhibit the highest and similar activity. The yield of 5,5′-(furan-2-ylmethylene)bis(2-methylfuran) can all reach 92% [43].

Through low-temperature pyrolysis and sulfonation reactions, humin obtained from glucose dehydration is transformed into a novel and effective carbonaceous solid acid catalyst. We studied a series of preparation conditions and discussed the structure function relationship of the obtained catalyst based on structural and compositional analysis. Compared with carbon catalysts derived from glucose, catalysts derived from humin have a more extensive surface area and greater density of SO_3_H groups, exhibiting higher catalytic activity and efficiency in the hydroxyalkylation/alkylation of 2-methylfuran and furfural (yield = 64.2% 323 K). In addition, the catalyst is capable of being reused for a minimum of four cycles without notable loss of activity, demonstrating good reusability [44].

Sodium-lignocellulosic- and lignosulfonate-derived aldehydes undergo phenolic condensation to form polymers, followed by proton exchange with sulfuric acid to form protonated sulfonic-acid-based catalysts. They are used as catalysts to catalyze the hydroxyalkylation/alkylation (HAA) of 2-methylfuran and furfural. LF resin prepared from sodium lignosulfonate and formaldehyde exhibited the most superior performance. LF resins showed greater activity and selectivity than amberlyst36 and amberlyst15 resins. Under optimized conditions, the yield of 5,5′-(furan-2-ylmethylene)bis(2-methylfuran) can reach 89% [45].

Preparation of hydrothermal carbon using biomass orange peel powder by means of the hydrothermal carbonization process. A variety of acidic catalysts were synthesized by functionalizing hydrothermal carbon with three distinct Brønsted acidic fractions, namely chlorosulfonic acid, nitric acid, and triethyl phosphate. The prepared catalysts are used to catalyze the hydroxyalkylation/alkylation reaction of 2-methylfuran and furfural under solvent-free conditions to form C_15_ fuel precursors. The effects of catalyst amount, time, and temperature on hydroxyalkylation/alkylation reactions were investigated. Under mild conditions, water carbon functionalized with sulfonic acid groups yields an optimal yield of 83% for C_15_ fuel precursor. The reaction between 2-methylfuran and acetone was also carried out using the best catalyst, resulting in a yield of 68% for 5,5′-(propane-2,2-diyl)bis(2-methylfuran) [46].

A series of aluminum-doped mesoporous silica spheres (Al MSS) catalysts with different Si/Al ratios and calcination temperatures were prepared and characterized. The prepared catalyst is used for the hydroxyalkylation/alkylation (HAA) of biobased furfural and 2-methylfuran to convert C_15_ diesel precursor. Among them, AlMSS20-450 (Si/Al = 20:1, calcined at 450 °C) showed the best activity and selectivity in the catalytic HAA reaction, with a yield of 94% within 20 min at 140 °C. The catalyst exhibits an increasing trend in product yield as the reaction temperature increases within the temperature range of 80 °C to 140 °C. The correlation between catalytic activity and surface acidic sites reveals that catalytic activity is mainly mediated by moderately and strongly acidic sites. Through poisoning analysis, it was found that Brønsted and Lewis acid sites synergistically catalyze the reaction, with the former being the main site [47]. The results of the hydroxyalkylation/alkylation reaction of 2-methylfuran and furfural are shown in Table 5.

Furfural (71%) was synthesized from rough corn cobs through acid hydrolysis dehydration, and 2-methylfuran (89%) was selectively hydrogenated on Raney nickel catalyst. C_15_ intermediate was prepared by hydroxyalkylation/alkylation reaction of 2-methylfuran and furfural catalyzed by formic acid and sulfuric acid. The yield of 5,5′-(furan-2-ylmethylene)bis(2-methylfuran) is 94.6% under sulfuric acid, while the yield of 5,5′-(furan-2-ylmethylene)bis(2-methylfuran) is 88.9% under formic acid [48].

Mesoporous/macroporous solid proton acids were prepared from sodium lignosulfonate (LS). These materials were used to for the solvent-free catalysis of the hydroxyalkylation/alkylation (HAA) of 2-methylfuran and furfural, cyclohexanone, butanal, acetone, α-angelica lactone, and levulinic acid. The yields of branched C_12_-C_16_ hydrocarbon precursors are distributed within 60% to 96%. In addition, the catalytic performance of carbon materials with high total amount (6–6.4 mmol·g^−1^) is superior to that of sulfonic acid resin (Amberlite^®^ IR120, Amberlyst^®^70, and zeolite, LS resin, and liquid acids (acetic acid, p-toluenesulfonic acid, and phenol). The turnover rate of the carbon catalyst (60LS40PS350H+) matches that of p-toluenesulfonic acid (186 h^−1^) in the conversion process of furfural. The catalytic activity and stability observed in LS-derived acidic carbon catalysts can be attributed to the robust Brønsted acidic SO_3_H groups that are covalently linked to their carbon framework, along with the hydrophilic surface functional groups (-COOH and -OH) that facilitate the adsorption of oxidation reactant molecules [49].

A series of MoO_3_-promoted mesoporous ZrO_2_ catalysts were prepared using zirconia isopropoxide, ammonium molybdate tetrahydroate, and pluronic P123 as raw materials. The HAA reaction between furfural and 2-methylfuran was studied using a newly developed mesoporous ZrO_2_ catalyst enhanced with MoO_3_. The influence of calcination temperature and the amount of MoO_3_ on the structural modifications and evolution of acidic sites in MoO_3_ enriched mesoporous ZrO_2_ catalysts was explained. The greatest acid density and superior catalytic performance were obtained at a loading rate of 20 wt% MoO_3_ and a calcination temperature of 873 K. At a molar ratio of 2:1, 323 K, and 5 h for under 2-methylfuran/furfural, with a catalyst of 1.25 g, the conversion rate of 2-methylfurfural was 85% [50].

Graphene oxide is very active in C-C coupling when it combines with Brønsted acidic oxygen functional groups and generates defect sites along highly oxidized surfaces and edges. On improved graphene oxide (IGO), hydroxyalkylation/alkylation (HAA reaction) of 2-methylfuran (2-MF) and furfural was carried out at a low temperature (60 °C) to obtain 95% selectivity of the C15 fuel precursor. The coupling of 2-MF with carbonyl compounds from C_3_ to C_6_ yields precursors with high carbon numbers ranged from 12 to 21 [51].

A one-pot method was developed to produce advanced diesel chain alkanes from biomass-derived furfural and 2-methylfuran (2-MF) using a multifunctional Pd/NbOPO_4_ catalyst via HAA and hydrogenation deoxygenation. Under the optimal reaction conditions, the total yield of alkanes in diesel fractions synthesized using the one-pot method using furfural and 2-methylfurfural as raw materials can reach 89.1% [52].

**Figure 5:**
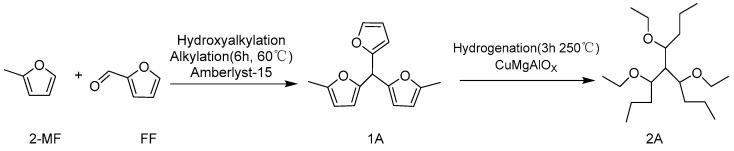
Route for the synthesis of ether compounds from 2-MF and FF [53].

In a fixed-bed reactor, Amberlyst-15 resin was used as a catalyst to convert 2-methylfuran and furfural into a high carbon polymer (**1A**). The reaction pathway is shown in Figure 5. Within 140 h, Amberlyst-15 exhibited good stability, with a conversion rate of over 70% for 2-methylfuran. Subsequently, the polymer was hydrogenated using CuMgAlOx catalyst (HDO) to produce ether compounds (**2A**), with a significant selectivity of 95.7% and carbon yield of 95.5% [53].

A series of SO_4_^2−^/TiO_2_ catalysts were synthesized using wetting impregnation method to catalyze the reaction of 2-methylfuran and furfural HAA to prepare C_15_ fuel precursors. The S/Ti-450 calcined at 450 °C exhibited the highest activity, with a yield of 97.9% for the C15 fuel precursor. The sufficient Brønsted acid sites and moderate Brønsted acid strength are the reasons why S/Ti-450 has strong catalytic performance. Stability studies have shown that S/Ti-450 can be regenerated. In addition, the HAA reaction of methylfuran with other carbonyl compounds (such as butanal, cyclohexanone, and acetone) was studied on S/Ti-450, and the required fuel precursor with high yield was obtained [54].

The scalable synthesis of SO_3_H-functionalized carbon from sugar industry waste (sugarcane bagasse) was reported. FT-IR and 13C-CP-MAS NMR were used to analyze the binding of SO_3_H functional groups on the carbon surface and the aromatic carbon framework. The total acidity of carbon synthesized by hydrothermal method is 5 mmol/g, and sulfur is evenly distributed on the surface of the carbon. The solvent-free HAA reaction of 2-methylfuran with various carbonyl compounds (furfural, 5-methylfurfural, 5-hydroxymethylfurfural, 5-Bromo furfural, vanillin, butanal, etc.) was catalyzed to produce excellent yields of fuel precursors at 60 °C [55].

The hydroxyalkylation/alkylation reactions of 2-methyluran (2-MF) with hydroxyacetone were studied on a range of solid acid catalysts (Nafion212, Nafion115, Amberlyst-36, Amberlyst-15, MC-SO_3_H, ZrP, H-β, H-ZSM-5 and H-USY). Among the catalysts studied, Nafion-212 showed the highest activity and stability. The yield of HAA product of 2-methylfuran and hydroxyacetone can reach 70.8%, Nafion-212 resin after reaction at 338 K for 6 h [56].

A new type of recyclable magnetic solid acid catalyst has been designed. [Fe_3_O_4_@SiO_2_-Pr-Py-H][2HSO_4_^2−^] was used for the HAA reaction of 2-methylfuran with formaldehyde. Under optimized conditions, the yield with catalyst, 2-methylfuran, formaldehyde, 65 °C, 3 h of bis(5-methylfuran-2-yl) methane can reach 86%. The results of the HAA reaction between 2-methylfuran and formaldehyde catalyzed by other solid acids are shown in Table 6. Under this reaction condition, 2-methylfuran undergoes HAA reaction were implemented with furfural, acetaldehyde, acetone, and benzaldehyde. The yield of the product from the HAA reaction of 2-methylfuran with aldehydes is 72%, and the yield of the product from the HAA reaction of 2-methylfuran with acetone is 65% [57]. The hydroxyalkylation/alkylation reaction results of 2-methylfuran and formaldehyde are shown in Table 6.

Using 2-methylfuran (2-MF) and a range of aldehydes for HAA reaction and HDO cascade catalytic process, long-chain alkanes with carbon range of C_11_-C_17_ are directly produced, with a total yield of 50–84%. The relative density of Lewis and Brønsted acidic sites in the catalyst significantly affects the catalytic activity and selectivity of HAA of 2-MF and formaldehyde. Sn/beta (12.5) exhibits significant Lewis acidity (including acid density and strength) and higher catalytic performance compared to other zeolites used in 2-MF and formaldehyde hydroxyalkylation/alkylation reactions. At 10 mmol 2-MF, Sn/beta (12.5), 5 mmol substrate, 100 °C, the yield of the hydroxyalkylation/alkylation products of 2-MF with formaldehyde, acetaldehyde, propanal, butanal, hydroxyacetaldehyde, furfural, and vanillin ranged from 58% to 92%. Even in aqueous solution, Sn/beta is reused at least six times under reaction conditions, with almost constant reaction activity [58].

The hydroxyalkylation/alkylation (HAA) reaction of angelica lactone with 2-methylfuran can be executed using a range of catalysts such as proton acids (Triflic acid, H_2_SO_4_, H_3_PO_4_, and acetic acid), Lewis acids (iron chloride, tin chloride, zinc chloride, and titanium chloride), and solid acids (Nafion212, Amberlyst15, and Amberlite IRC 76CRF). All three acids exhibit good activity in the hydroxyalkylation/alkylation (HAA) reaction of 2-methylfuran angelica lactone with 2-methylfuran. As the acid strength increases, the reaction activity also increases. Under optimized conditions (323 K; 1 h), the yield of 4,4-bis (5-methyluran-2-yl) pentanoic acid can reach 81.3% [59].

The binding of formic acid and Sn Mont provides a method for producing liquid fuel precursors of C_15_ and C_21_ through the hydroxyalkylation/alkylation reaction of 2-methylfuran and carbohydrates. The combination of formic acid and Sn Mont provides a liquid fuel precursor for the production of C_15_ and C_21_ from carbohydrate and 2-methylfuran hydroxyalkylation/alkylation reactions. Sn Mont glucose efficiently isomerizes to fructose at its Lewis acid site. In addition, when formic acid is present, it promotes the dehydration of fructose formed in situ into HMF, which then condenses with MF. Formic acid plays various roles as a powerful Brønsted acid catalyst and as a solvent for dissolving carbohydrates. A 40% yield of 5,5′-((5-(5-methyluran-2-yl) methyl) furan-2-yl) methylene)bis(2-methyluran) was obtained by reacting sucrose as the raw material. In the case of fructose substitution, the yield of 5,5′-((5-(5-methyluran-2-yl) methyl) furan-2-yl) methylene)bis(2-methylfuran) is 54%. This new green approach offers a novel path toward the sustainable development of biomass to add value to liquid hydrocarbon precursors and chemicals [60].

A simple and novel silica-supported, sulfonic-acid-functionalized isonicotinic acid catalyst was prepared by treating isonicotinic acid with chlorosulfonic acid and then conducting a multiphase reaction on silica (SO_3_H-INA@SiO_2_). This catalyst catalyzes the solvent-free conversion of 2-methylfuran to diesel fuel precursors of C_15_ and C_20_ units. Under optimized reaction conditions, over SO_3_H-INA@SiO_2_ complete conversion of 2-methylfuran, the yields of 5,5-bis (5-methylfuran-2-yl) pentane-2-one (**1**) and 2,4,4-tris (5-methylfurfural-2-yl) penta-1-ol (**2**) are 19% and 67%, respectively [61]

3-Bromopyridine heterogeneous phosphotungstic acid (3-BrPyPW) was prepared using a solvothermal method and used for hydroxyalkylation/alkylation reactions of 5-hydroxymethylfurfural (HMF) and 2-MF. Under the optimal reaction conditions (1 mmol HMF and 3 mmol 2-MF at 100 °C for 8 h), the conversion rate of 2-MF was 82.0%, and the MMBM yield reached 57.1%. The 3-BrPyPW catalyst can be reused four times without a significant decrease in activity, and its structure remains unchanged after recovery [62].

### Hydroxylalkylation/Alkylation Reaction of Methylfuran and Cyclopentanone

The solvent-free hydroxyalkylation/alkylation (HAA) reaction of 2-MF and cyclopentanone is carried out on a range of acid catalysts (nafion212, 72% H_2_SO_4_, amberlyst-15, amberlyst-36, H-USY, ZrP, H-β and H-ZSM-5). In the hydroxyalkylation/alkylation reaction of 2-MF and cyclopentanone, two products, 5,5-bis(5-methylfuran-2-yl)pentan-2-one and 5,5′-(cyclopentane-1-diyl)bis(2-methylfuran), are generated. 5,5′-(cyclopentane-1,1-diyl)bis(2-methylfuran) is a product of direct hydroxyalkylation/alkylation reaction between cyclopentanone and methylfuran, and 5,5-bis(5-methylfuran-2-yl) pentan-2-one is a product of 4-oxopentanal produced by the hydrolysis of methylfuran and the hydroxyalkylation/alkylation reaction of two molecules of methylfuran. Among the studied catalysts, Nafion212 showed superior activity, with a combined yield of 5,5-bis(5-methylfuran-2-yl)pentan-2-one and 5,5′-(cyclopentane-1-diyl)bis(2-methylfuran) reaching 95% [63].

Hydrophobic mesoporous sulfonic acid (PS) and fluorosulfonic acid (PCS) resins were achieved through simple solvothermal reactions and ion exchange treatments. They possess excellent mesoporous structures and specific surface areas ranging from 300 to 700 m^2^⸳g^−1^. Valuable acidic PS and PCS catalyze biomass driven 2-methylfuran with cyclic ketones (cyclohexanone and cyclohexanone) for hydroxylation/alkylation. It is worth noting that compared to the extensively used Amberlyst-15 and Nafion-212, PS and PCS exhibit superior hydrophobicity and lipophilicity. In the biomass driven cyclic ketones (cyclohexanone and cyclohexanone) with 2-methylfuran hydrogenation/alkylation, PS exhibits superior activity and selectivity compared to Amberlyst-15, whereas PCS shows greater catalytic performance and selectivity than Nafion-212. In addition, both PS and PCS demonstrate good stability over a sequence of five consecutive runs [64].

A new type of hydrophobic aromatic sulfonic acid functionalized biochar was successfully synthesized by using amino aromatic sulfonic acid (such as 4-aminobenzenesulfonic acid, 4-amino-benzenesulfonic acid, 4-amino-3-hydroxy-1-naphthalenesulfonic acid, 8-amino-1-naphthalenesulfo-nate) to reduce biochar in one pot of diazotization. It boasts a substantial specific surface area within the range of 200 to 400 m^2^⸳g^−1^, a hydrophobic network with a water contact angle exceeding 120°, and a sulfonic acid concentration higher than 1.0 mmol/g. In addition, as the length of aromatic hydrocarbons and the amount of aromatic sulfonic acid grafting increase, the hydrophilicity and acidity also increase. In the process of alkylating 2-methylfuran with cyclopentanone, it is used to produce high-density biofuels. Due to its hydrophobicity and strong acidity, its catalytic efficiency, with a target product yield of 76.1%, surpasses that of Amberlyst-15, which has a yield of 50.2% and also exceeds traditional sulfonated biochar SO_3_H at 13.2%. In addition, the catalyst showed no significant decrease in catalytic performance over six cycles of operation, indicating good stability of the catalyst. Successfully preparing hydrophobic biochar based acidic catalysts presents a fresh strategy for the high-value utilization of biochar while also counteracting the detrimental influence of water on many catalytic reactions [65].

Biochar functionalized with a hydrophobic sulfonic acid with a BET surface area within the range of 400 to 700 m^2^⸳g^−1^, a sulfonic acid density higher than 2.0 mmol/g, and a 136° water contact angle was synthesized through a two-step diazotization technique. The process involved the use of benzenesulfonic acid and tert-butylbenzene, with 4-aminobenzenesulfonic acid as the sulfonating agent and 4-tert-butylphenylamine contributing the hydrophobicity. In contrast to amberlyst-15 and H_2_SO_4_-sulfonated biochar, hydrophobic acidic biochar demonstrates superior activity and selectivity in the alkylation of 4-ethylphenol with benzyl alcohol and the hydroxyalkylation reaction of 2-methylfuran with cyclohexanone (cyclohexanone and cyclopentanone), making it suitable for the production of high-density biofuels. In addition, they demonstrate reliable stability and are capable of being reused at least five times. The work delivers a powerful catalytic system for the preparation of high-density biofuels and also presents a fresh strategy for biochar enhancement through diazo grafting method [66].

The catalytic HAA reaction for the production of high-density biofuel precursors (FCF) from hemicellulose-derived 2-methylfuran and lignin-derived cyclohexanone was studied over a range of acidic catalysts. The yield of 5,5′-(cyclohexane 1-diyl)bis(2-methylfuran) on superacid Nafion 212 film is as high as 89.1%. After hydrodeoxygenation, the class obtains high-density biofuels [67].

Two catalysts TBA_4_[SiW_11_O_39_(O(SiC_3_H_6_SO_3_H)_2_)] (compound **2**) and TBA_4_[SiW_11_O_39_ (O(SiC_8_H_8_SO_3_H)_2_)] (complex **3**) were successfully synthesized by covalently grafting different sulfonic acid (-SO_3_H) groups onto [SiW_11_O_39_] 8-clusters. The strong Brønsted acids of compounds **2** and **3** were characterized by potentiometric titration, pyridine adsorption studies, and 31P trimethylphosphine oxide (TMPO) nuclear magnetic resonance (NMR). When applied to the hydroxyalkylation/alkylation (HAA) reaction of 2-methylfuran (2-MF) and cyclohexanone, compound **2** exhibits better catalytic performance than compound 3, with a conversion rate of about 93%, a yield of 79.9% for the single ring fuel precursor (**1a**), and a selectivity of 85.7%. Finally, compound **2** also exhibited excellent catalytic activity in the HAA reaction between 2-MF and biomass-derived carbonyl compounds such as furfural, 5-methylfurfural, acetone, butanal, and 4-methoxybenzaldehyde [68].

A novel solid acid catalyst was prepared using paraformaldehyde, chlorosulfonic acid, and bisphenol A as raw materials and employed in the HAA reaction of 2-methylfuran (2-MF) and cyclohexanone. After the reaction conditions were optimized, the conversion rate of 2-MF hit 99%, and the yield of 5,5′-(cyclohexane-1,1-diyl) bis(2-methylfuran) was 98%. The resin exhibits higher activity and catalytic efficiency compared to Amberlyst 15 resin and possesses a higher acid strength. Meanwhile, the catalyst underwent characterization through acid-base titration and infrared spectroscopy. The HAA product of cyclopentaenone and 2-methylfuran was further converted to aviation kerosene by hydrodeoxygenation (HDO) over Ni/SiO_2_ catalyst prepared using the wet method at 93% yield [69].

**Figure 6:**
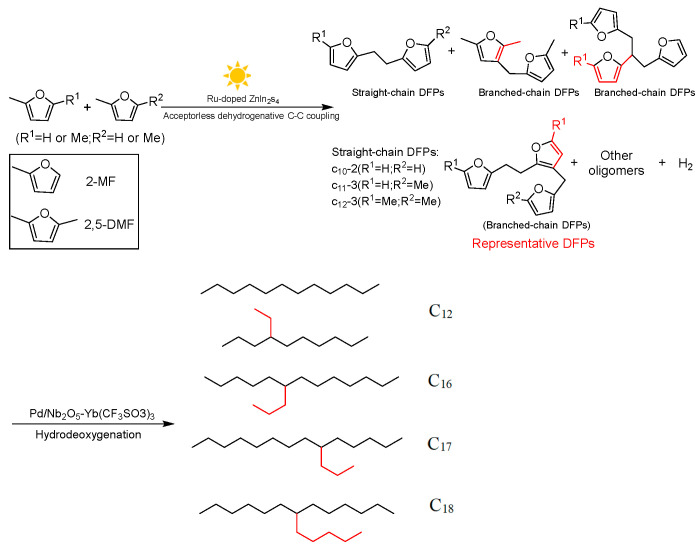
Chemical reactions involved in conversion of 2,5-DMF/2-MF into diesel fuel [70].

The apparent quantum efficiency (AQY) of the Ru-doped ZnIn_2_S_4_ catalyst is 15.2%, visible light was used to couple 2,5-dimethylfuran(2,5-DMF) derived from lignocellulose and 2-methylfuran dehydrogenation to H_2_ and DFP. Photogenerated holes oxidize the furfural C-H bonds of 2,5-DMF/2-MF, transfer protons and furfural radicals, and form the required DFP through C-C coupling. Meanwhile, the proton is converted into H_2_ through the electrons that are produced. Following the HDO reaction, DFP is transformed into~C_10_-C_18_ alkanes composed of linear and branched alkanes, resulting in a diesel fuel that is similar to petroleum diesel in hydrocarbon content [70]. The chemical reaction pathway involved in converting 2,5-DMF/2-MF into diesel fuel is shown in Figure 6.

**Figure 7:**
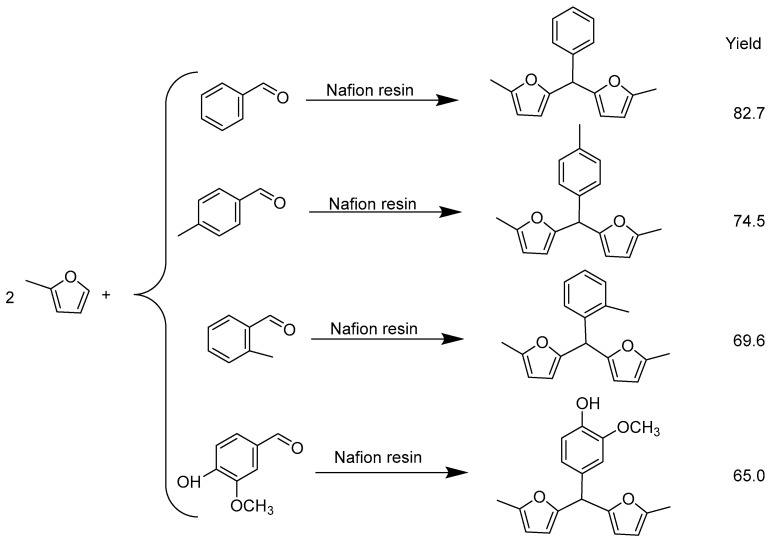
Results for the HAA of 2-MF with lignocellulose-derived aromatic aldehydes [71].

5,5′-(phenylmethylene)bis(2-methylfuran) was synthesized through hydroxyalkylation/alkylation (HAA) of 2-methylfuran and acid catalyzed by benzaldehyde. Among the liquid acid and solid acid resins studied, Nafion resin exhibited the highest HAA activity for the reaction betweenbenzaldehyde and 2-methylfuran, which is consistent with its highest acid strength.Under optimized conditions on Nafion resin, the yield of 5,5′-(phenylmethylene)bis(2-methylfuran) can reach 82.7% [71]. The HAA of 2-MF and lignocellulose-derived aromatic aldehydes is shown in Figure 7.

## 3. Acetylation Reaction of Methylfuran

**Figure 8:**
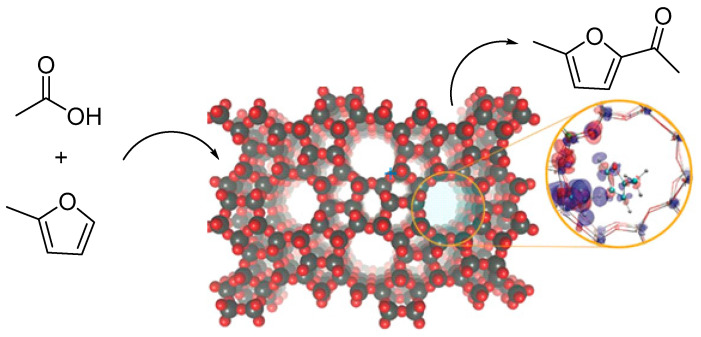
Direct acylation of 2-MF with acetic acid over HZSM-5 [72].

Methylfuran was directly acylated with biomass-derived acetic acid over HZSM-5 in the presence of water. This direct coupling restricts the hydroxyalkylation reaction of furan compounds in the production of acetylmethylfuran. Density functional theory calculations and reaction kinetics indicate that the apparent energy barriers are involved in the dehydration of acids to create surface acyl species, derived from calculations, are similar to those measured experimentally, suggesting that this step is crucial in establishing the net rate of the reaction. Water suppresses the overall rate but does not affect the selectivity of acylation products. The Furan species efficiently stabilize the charges present at the transition state, thereby reducing the entire activation energy barrier. The results indicate that producing higher value products from biomass is a promising new pathway for the formation of C-C bonds [72]. The schematic diagram of the direct acylation reaction between acetic acid and methylfuran is shown in Figure 8.

**Figure 9:**
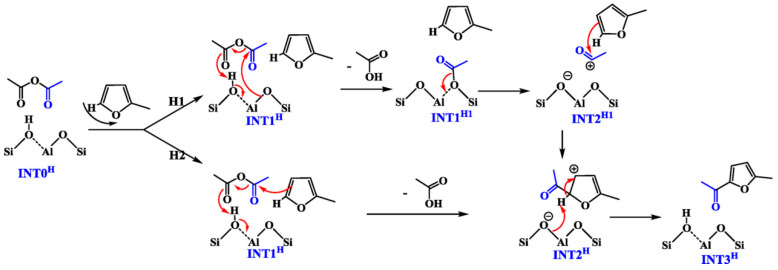
Stepwise (H1) and concerted (H2) mechanisms for the acylation of methylfuran on H−[Al]−Beta [73].

The acylation reaction of acetic anhydride and methylfuran was studied using Lewis and Brønsted acidic zeolite catalysts. The acylation reaction rate was highest on a per-gram basis for Beta zeolites with high aluminum contents (Si/Al = 23), while the highest turnover frequency per metal site was observed in Beta zeolites with low aluminum contents (Si/Al = 138). In the Lewis acid zeolite, the turnover frequency of [Sn]-β is higher than that of [Hf]-, [Zr]-, and [Ti]-β. For [Al] with different Si/Al ratios-β, Similar apparent activation energies have been discovered. Calculations show that in [Al]- and [Sn]-β, the rate of methylfuranylation is governed by the dissociation of the anhydride C-O-C bond and that the elimination of hydrogen is the rate-determining step for furanylation [73]. The mechanism of methylfuran acylation on H-[Al]-Beta is shown in Figure 9.

**Figure 10:**
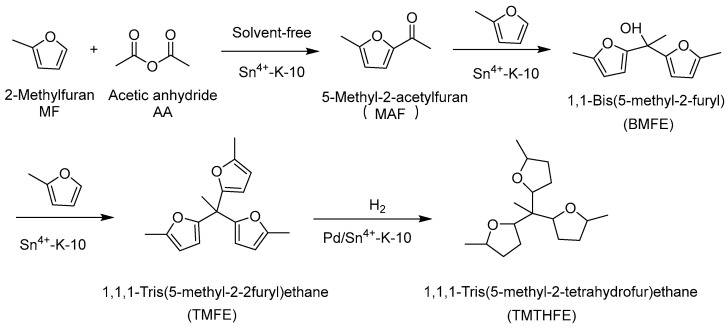
Catalytic synthesis of C_17_ oxygenate (TMFE) from biomass-derived MF and AA over Sn-exchange K-10 clay via cascade acylation–alkylation, followed by hydrogenation over Pd/Sn^4+^-K-10 to give TMTHFE [74].

Under solvent-free conditions, one-pot cascade acylation and hydroxyalkylation/alkylation of biomass-derived acetic anhydride and 2-methylfuran was carried out on Sn^4+^-exchanged K-10 montmorillonite (Sn^4+^-K-10) to produce C_17_ oxygen-containing compound 1,1,1-tris (5-methyl-2-furanyl) ethane. The yield of 1,1,1-tris (5-methyl-2-furanyl) ethane is 87% and the selectivity is 90% within 8 h at 60 °C. The characterization results indicate that Sn^4+^-K-10 features a layered, mesoporous texture of 5.7 nm, coordinated Lewis–Brønsted acid coordination sites, and homogeneously dispersed Sn species, all of which contribute significantly to the major reactivity and recyclability of Sn^4+^-K-10 [74]. The acylation–alkylation reaction route of methylfuran and acetic anhydride on Sn-exchange K-10 clay is shown in Figure 10.

Under the catalysis of montmorillonite treated with different concentrations of hydrochloric acid, C_17_ diesel precursor 1,1,1-tris (5-methyl-2-furanyl) ethane (TEMF) was synthesized through a cascade acetylation and hydroxyalkylation/alkylation reaction of biobased 2-methylfuran (MF) and acetic anhydride (AA). Under optimized conditions (K-10–1, 6 h and 40 °C), the yield of 1,1,1-tris (5-methyl-2-furanyl) ethane can reach 70%. We studied the catalytic mechanisms of cascade reactions in different types of acids (Brønsted acid and Lewis acid). Weak Brønsted acids and Lewis acids predominantly facilitate hydroxyalkylation and acetylation reactions, while strong Brønsted acids are chiefly responsible for the subsequent alkylation steps [75].

**Figure 11:**
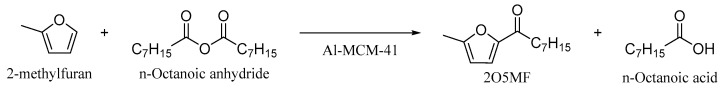
n-octanoic anhydride (C_8_) reacts with 2-methylfuran in solvent heptane to form 2-octanoyl-5-methylfuran(2O5MF) and n-octanoic acid in the presence of a Al-MCM-41 [76].

The Friedel–Crafts acylation of long-chain fatty acid derivatives with bio-derived furans is essential for producing alkylfuran, a key precursor in the creation of bio-renewable furan-based surfactants. Al-MCM-41(a mesoporous aluminosilicate) was utilized to study the steady-state kinetics and reaction mechanism of the acylation of 2-methylfuran with n-octanoic anhydride. The formation of acylation product 2-octyl-5-methylfuran (2O5MF) achieved apparent activation energy (15.5 ± 1.4 kcal mol^−1^) in the temperature range of 348–408 K. At the concentrations of 2-methylfuran and anhydride, the apparent reaction rates are approximately ~0.6 and ~0.5, respectively, while the apparent rate orders measured at the product concentration were close to zero, indicating that the inhibitory effect of the product can be ignored. An Eley–Rideal mechanism for catalytic acylation was suggested to account for the observed apparent rate order in the experiment [76]. The pathway for the reaction of n-octanoic anhydride (C_8_) with 2-methylfuran to generate 2-octyl-5-methylfuran is shown in Figure 11.

## 4. Diels–Alder Reaction of Methylfuran and Olefin

### 4.1. Diels–Alder Reaction of 2-Methylfuran and Ethylene, Propylene

**Figure 12:**
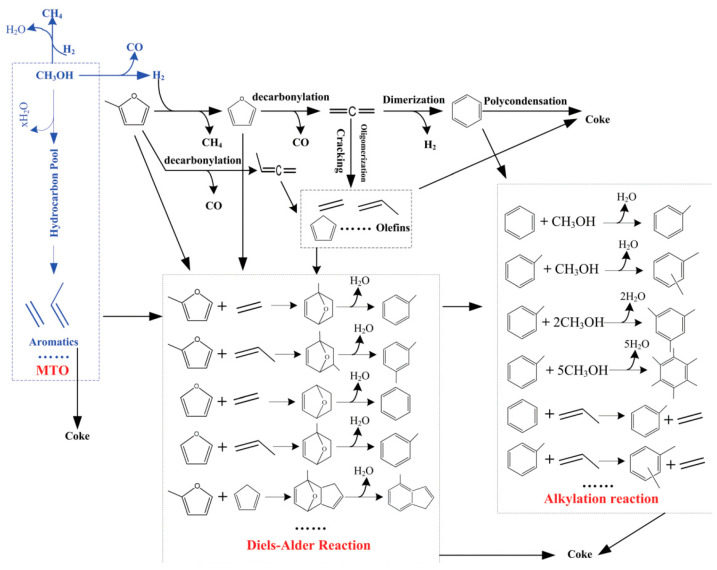
The pathway of converting MF and methanol into aromatics [77].

Biomass-derived furan and methanol are coupled and converted to produce aromatics in a continuous fixed-bed reactor, with ZSM-5 serving as a catalyst in the fixed-bed reactor. The influence of the methanol to MF (2-methylfuran) molar ratio, space velocity (WHSV), and reaction temperature on the conversion product distribution of MF and methanol coupling were studied. The experimental data reveal that coupling MF with methanol results in aromatic yields 5.2 times greater than those obtained from the rapid pyrolysis of MF by itself under catalysis. In addition, it improves the selectivity of xylene and the yield of olefins and reduces the formation of coke. There is an important synergy between methanol and MF. The synergistic impact is thought to result from the reaction between olefins and methanol, the Diels–Alder reaction between olefins and furan, and the alkylation reaction between methanol and benzene/toluene during the coupling conversion of MF and methanol. The molar ratio of methanol to MF is 2 at 550 °C, which is the reaction condition for maximizing the synergistic effect. In addition, this study also expanded the comparative investigation of the coupling transformation of bio-derived furans (MF, 2,5-dimethylfuran, furfuryl alcohol, and furfural) with methanol. The reaction pathway is shown in Figure 12. The coupling conversion of DMF with methanol yielded the highest aromatics, olefins, and the lowest coke, indicating that DMF is the optimal choice for the coupling transformation of bio-derived furans with methanol to aromatics [77].

**Figure 13:**
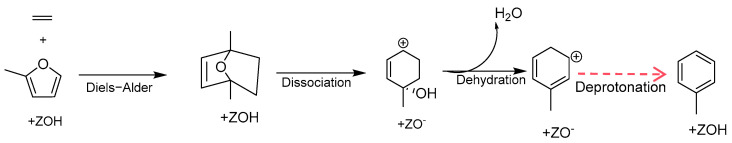
The possible Diels−Alder cycloaddition route of ethylene and 2−methylfuran to aromatics over the ZSM-5 zeolites [78].

On ZSM-5 containing Sn, 2-methylfuran (2-MF) undergoes a graded co aromatization reaction with methanol at 500 °C. The data suggest that Sn-enriched catalysts exhibit higher aromatics selectivity compared to non-Sn catalysts, because the increase in Lewis acidic sites resulting from Sn doping facilitates the Diels–Alder (DA) reaction between olefins and 2-MF. Modify tin containing ZSM-5 through alkaline treatment to form a graded structure. The creation of new mesopores enhances the rate of mass transfer, improve coke resistance, and greatly enhance the selectivity and catalytic stability of aromatics. In addition, the formation routes of aromatic hydrocarbons were systematically explored through isotope experiments and theoretical calculations. The results indicate that during the co feeding process of ^13^C methanol and 2-MF, the individual conversion of 2-MF, olefin/aromatic methylation, and DA reaction may occur simultaneously, however, individual transformations of ^13^C methanol reaction appears to be partially suppressed. Calculations indicate that the cycloaddition of furan and olefins is a rate determining step for the formation of aromatics through the Diels–Alder pathway [78]. The route for synthesizing aromatic compounds through the cycloaddition reaction of methylfuran (dimethylfuran) and ethylene is shown in Figure 13.

**Figure 14:**
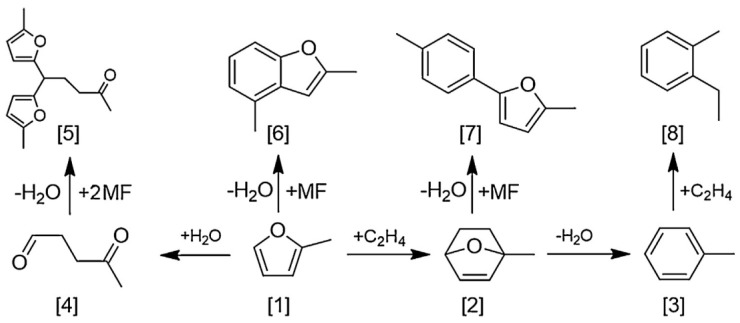
Reaction of 2−methylfuran and ethylene to toluene in H−form zeolites. Molecules: [1] 2−methylfuran (MF), [2] MF/C_2_H_4_ cycloadduct, [3] toluene, [4] 4−oxopentanal, [5] 2−methyfuran trimer, [6] 2−methylfuran dimer, [7] polymer product, and [8] alkylation product [79].

The Diels–Alder cycloaddition of biomass-derived ethylene and 2-methylfuran to generate renewable toluene is achieved through thermochemical pathways. The kinetics and reaction pathway of Diels–Alder cycloaddition of ethylene and 2-methylfuran to toluene were evaluated using H-BEA and Sn-BEA catalysts. The kinetic analysis of chemical reactions revealed the rate control steps of Diels–Alder cycloaddition and dehydration reactions. The yield of Diels–Alder cycloaddition reaction between ethylene and 2-methylfuran is independent of the concentration of Brønsted acid, while the dehydration rate of Diels–Alder cycloaddition complex is related to the Brønsted acid concentration. The selectivity for toluene has never exceeded 46%, as the by-product reaction consumes 2-methylfuran, including dimerization of 2-methylfuran, hydrolysis of 2-methylfuran, formation of trimers after ring opening, and incomplete dehydration of Diels–Alder ring adducts of 2-methylfuran and ethylene [79]. The possible pathways for the 2-methyluran and ethylene cycloaddition reaction are shown in Figure 14.

Various Lewis acid metal chlorides, cation-exchanged Y zeolite, Sn, and Zr-containing β exploring the correlation between acidic sites and the Diels–Alder activity of 2-methylfuran with ethylene convert to toluene in zeolites. Compared with the Brønsted acidic zeolites of H-Y and H-Beta, the Lewis acid catalysts of Na-Y and AlCl_3_ zeolites exhibit excellent selectivity for toluene production. The high selectivity of toluene is mainly attributed to the ability of Lewis acid to delay side reactions (such as alkylation and oligomerization) and speed up the cycloaddition of ethylene and MF. Under the conditions of Brønsted acidic zeolites, side reactions are notably increased, leading to a decrease in selectivity for toluene. Lewis acid AlCl_3_ produces a maximum toluene yield of 70% after 24 h at 250 °C. Na-Y also has stronger Lewis acidic site activity than Zr-Beta and Sn-with Lewis acidic sites, it is proved to be superior in catalytic Diels–Alder cascade reaction and MF dehydration aromatization reaction. Under optimal reaction conditions, under an almost-complete 2-methylfuran conversion rate (>96%), Na-Y can achieve a toluene yield of 65% [80].

### 4.2. Diels–Alder Reaction of 2-Methylfuran and Dicyclopentadiene

**Figure 15:**
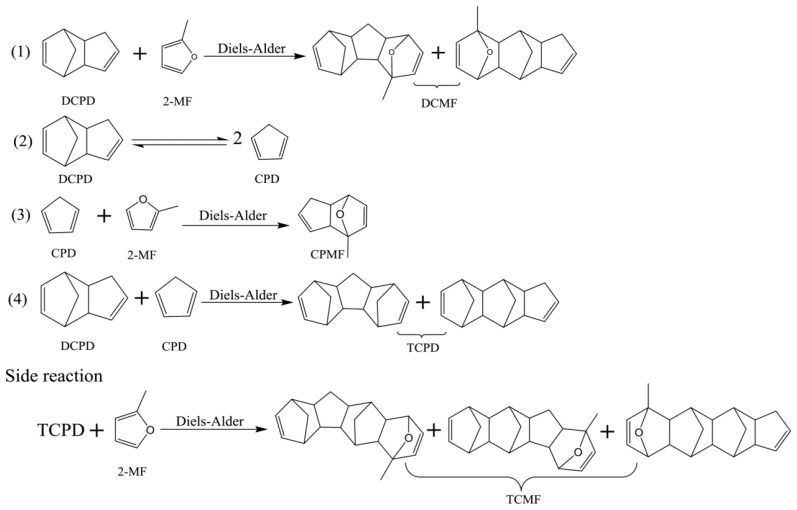
Reaction pathway for Diels–Alder reaction of 2-MF and DCPD [81].

A high-density liquid fuel was synthesized from 2-methylfuran from lignocellulose and dicyclopentadiene (DCPD) from petroleum by Diels–Alder reaction, followed by hydrodeoxygenation. Examine the effects of reaction temperature, catalyst, and reactant ratio on the product distribution of Diels–Alder reaction. In comparison to the other catalysts studied, zeolite demonstrated superior catalytic activity and exhibited good recyclability. On HY zeolite, high reactant conversion and acceptable target product selectivity were achieved at 150 °C and a 2:1 2-MF/DCPD ratio. After hydrogenation and deoxygenation, the obtained fuel density is 0.984 g/mL, much higher than the widely used JP10 fuel has a density of 0.984 g/mL. It also features a low freezing point of −58 °C and provides a net combustion heat of 41.96 MJ/L [81]. Reaction pathway for Diels–Alder reaction of 2-MF and DCPD was shown in Figure 15.

### 4.3. Diels–Alder Reaction of 2-Methylfuran and Norbornene

**Figure 16:**
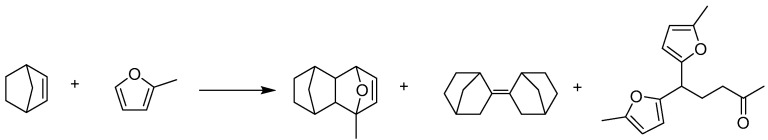
Reaction pathway for Diels–Alder reaction of 2-MF and norbornene [82].

JP-10 analog fuel was synthesized through a one-pot Diels–Alder/hydrogenolysis deoxygenation reaction using petroleum-derived dicyclopentadiene and bio-derived furans as feedstocks. Si/Al ratio of 25 Hβ with zeolite shows the the highest catalytic efficiency in the Diels–Alder reaction with a yield of 75.9%, attributed to its large surface area and high acid concentration, notably the Brønsted/Lewis acid site ratio. Dynamics studies have shown that the apparent activation energy of the Diels–Alder reaction between norbornene and 2-methylfuran is 45.67 kJ/mol, which is significantly lower compared to the dimerization reaction of norbornene (52.48 kJ/mol) and the trimerization reaction of 2-methylfuran (63.61 kJ/mol). Importantly, synthetic fuels have higher density and volumetric net heat of combustion as well as better low-temperature viscosity. The study offers an effective and promising approach for the production of high-performance JP-10 fuel substitutes through co-conversion of biological and petroleum derivatives [82]. Reaction pathway for Diels–Alder reaction of 2-MF and norbornene was shown in Figure 16.

### 4.4. Diels–Alder Reaction of 2-Methylfuran and Maleic Anhydride

**Figure 17:**
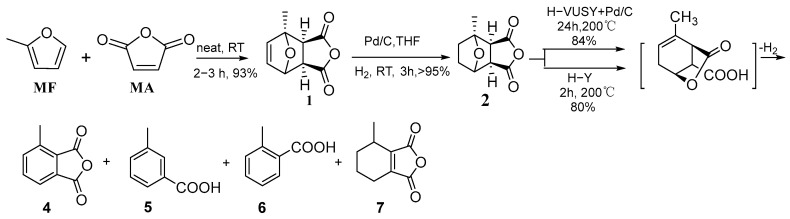
The cycloaddition reaction pathway of methylfuran and maleic anhydride [83].

Aromatic compounds from renewable sources can be efficiently created from furans by incorporating a hydrogenation step as an intermediary in the Diels–Alder (DA) aromatization process, which effectively inhibits the anti-Diels–Alder activity. The aromatization of hydrogenated DA adducts to produce toluene requires the use of solid acid dehydration catalysts and metal-based dehydrogenation catalysts in series catalysis. In solid-phase reactions using only acidic zeolites, without solvents or dehydrogenation catalysts and with short reaction times, hydrogenated Diels–Alder adduct can be easily transformed into renewable aromatics with selectivities of up to 80%. The diene/dienophilic combination of (methylated) furan and the hydrogenation complex of maleic anhydride are effectively converted into renewable aromatic hydrocarbons through this innovative route. H-Y zeolite has the best performance and is easy to reuse after calcination [83]. The cycloaddition reaction pathway between 2-methylfuran and maleic anhydride is shown in Figure 17.

**Figure 18:**
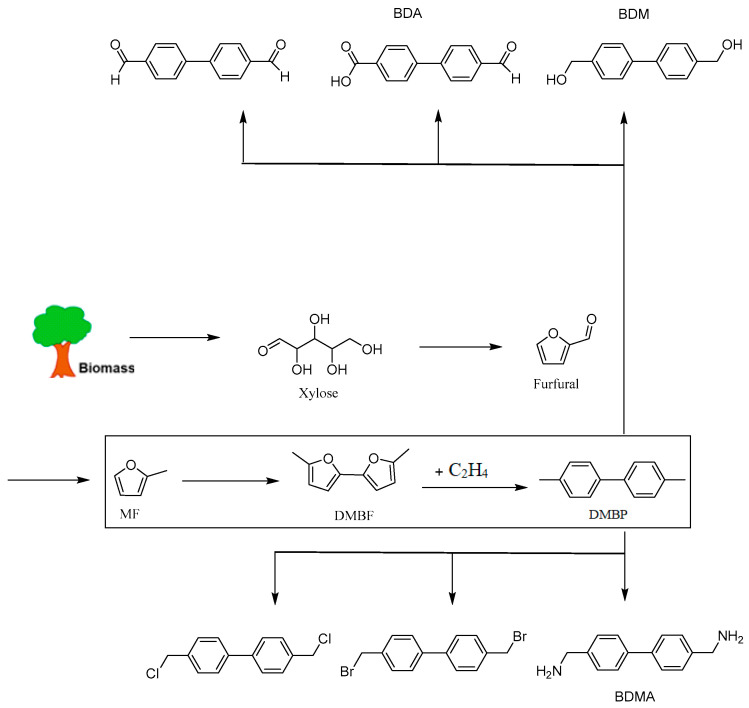
Reaction pathway for the synthesis of 4,4′-dimethylbiphenyl (DMBP) from biomass-derived 2-methylfuran (MF) via the formation of 5,5′-dimethylbifuran (DMBF), and the potential functionalization of DMBP into valuable platform chemicals including 4,4′-biphenyldicarboxylic ccid (BDA), 4,4′-biphenyldiyldimethanol (BDM) and 4,4′-biphenyldiyldimethanamine (BDMA) [84].

2-methylfuran (MF) is a two-step process for producing 4,4′-dimethylbiphenyl (DMBP) by forming the intermediate 5,5′-dimethyl-2,2′-difuran (DMBF). With trifluoroacetic acid (TFA) present, Pd acts as a catalyst for the oxidative coupling of MF to DMBF, achieving high selectivity at 94%. High O_2_ pressure (7 bar) and high TFA concentration (3M) are crucial for improving the rate of DMBF formation. Silicon dioxide supported phosphoric acid (PSiO_2_) catalyzes the series Diels–Alder and dehydration reactions of DMBF and ethylene to produce DMBP (83% yield). The high yield and selectivity obtained for DMBP are due to the weak Brønsted acid sites characteristic of P-SiO_2_, which dehydrates the furan ethylene adduct without forming carbon deposits [84]. The synthetic route to 4,4-dimethylbiphenyl derivatives through the coupling of methylfuran and ethylene cycloaddition is shown in Figure 18.

## 5. Conclusions

2-methylfuran is mainly prepared through processes like hydrolysis, dehydration, and selective hydrogenation of biomass hemicellulose. Due to its special physical and chemical properties, 2-methylfuran can be used as a raw material and intermediate in the production of anti-malaria drugs, and its combustion efficiency is high. It can be mixed with gasoline to reduce gasoline consumption. 2-methylfuran can undergo hydroxyalkylation/alkylation reactions with aldehydes, ketones, and esters derived from biomass to grow carbon chains and then undergo hydrogenation deoxygenation to produce alkane fuels. The aldehydes involved in this article include butyraldehyde, furfural, 5-hydroxymethylfurfural, 5-methylfurfural, benzaldehyde, vanillin, glutaraldehyde, propanal, acetaldehyde, and formaldehyde. The ketones involved include acetone, hydroxyacetone, butanone, 2-pentanone, cyclopentanone, cyclohexanone, and isopropylacetone. The esters involved include ethyl acetate and angelolactone. The catalysts used in hydroxyalkylation/alkylation reactions can be liquid acids, solid acids, or ionic liquids. Liquid acids mainly comprise sulfuric acid and p-toluenesulfonic acid. The solid acids involved include cation exchange resins, zeolite molecular sieves, oxides, and sulfonic-acid-based, carbon-based solid acids. Cation exchange resins include Nafion212, Nafion115, Amberlyst-15, Amberlyst-36, and Amberlyst^®^IR120, Amberlyst^®^70. Molecular sieves include USY, Beta, MCM-41, ITQ-2, ZrP, H-Y, HZSM5, Sn/Beta, Sn/Mont, Al mss20-450, [Fe_3_O_4_@SiO_2_-Pr-Py-H][2HSO_4_^2−^], etc. Oxides include MoO_3_ and ZrO_3_. Sulfonic-acid-based, carbon-based solid acids include Dowex50wx2-100, PTNT, CMK-3-SO_3_H, MC-SO_3_H, Ac-SO_3_H, LF, PS, PCS, SHC, HC573-S423, LS-RES, and 80LS20PS350H^+^.

2-methylfuran can undergo acylation reactions with acetic acid (or acetic anhydride) and n-octanoic anhydride on solid acid catalysts to produce 1-(5-methylfuran-2-yl) ethane-1-one and 1-(5-methylfuran-2-yl) non-1-one, respectively. It can be used as a raw material for surfactants.

2-methylfuran can undergo cycloaddition reactions with ethylene, dicyclopentadiene, norbornene, and maleic anhydride to form monocyclic or polycyclic compounds, which can be used as fine chemicals and high-density fuels. Palladium catalyzed oxidative coupling of 2-methylfuran under trifluoroacetic acid conditions produces 5,5′-dimethylfuran, which then undergoes cycloaddition with ethylene to form 4,4′-dimethylbiphenyl. In one class, the sulfonic acid functionality is bonded in a direct manner to the imidazole ring, while in the other, the sulfonic acid functionality is separated by an alkyl linker.

The future application of 2-methylfuran can be considered from two aspects. On one hand, 2-methylfuran can be prepared into high-density aviation fuel through Diels–Alder cycloaddition, hydroxyalkylation/alkylation reactions. On the other hand, the products obtained from the reaction of 2-methylfuran with oxygenated compounds can be used to develop surfactants, pharmaceutical intermediates, perfumes, essence, etc.

## Figures and Tables

**Table 1:** Synthesis of 2,2′,2″-methylidenetris [5-methylfuran] from 5-methylfurfural and 2-methylfuran.

Entry	Molar Ratio 2-MF/5-Methylfurfural	Catalyst	Mass Ratio p-TosOH/2-MF	Time (h)	Temperature (°C)	Yield (%)	Reference
1	5.0	p-TosOH	0.0230	6	50	93	[33]
2	5.0	p-TosOH	0.0350	6	50	93	[34]
3	5.0	Sulfuric acid	0.2449	6	50	83	[34]

**Table 2:** Synthesis of 2,2′-butylidenebis (5-methylfuran) from butanal and 2-methylfuran.

Entry	Molar Ratio 2-MF/Butanal	Catalyst	Mass Ratio p-TosOH/2-MF	Time (h)	Temperature (°C)	Yield (%)	Reference
1	2.0	p-TosOH	0.0324	6	50	79	[33]
2	3.0	p-TosOH	0.0222	6	50	84	[33]
3	3.5	p-TosOH	0.0192	6	50	88	[33]
4	2.0	Amberlyst-15	0.0331	22	50	59	[33]
5	3.0	Amberlyst-15	0.0222	22	50	69	[33]
6	2.0	USY	0.0167	8	50	53	[35]
7	2.0	Beta(comm)	0.0167	8	50	67	[35]
8	2.0	Beta(nano)	0.0167	8	50	59	[35]
9	2.0	Beta(OH^−^)	0.0167	8	50	34	[35]
10	2.0	Beta(F^−^)	0.0167	8	50	16	[35]
11	2.0	MCM-41 (Si/Al ratio = 15)	0.0167	8	50	45	[35]
12	2.0	MCM-41(Si/Al ratio = 28)	0.0167	8	50	60	[35]
13	2.0	ITQ-2	0.0167	8	50	86	[35]
14	2.0	Dowex 50WX2-100	0.0167	8	50	80	[35]
15	2.0	Nafion212	0.0915	2	50	89.5	[36]
16	2.0	PTNT	0.0457	6	50	77	[37]

**Table 3:** Hydroxyalkylation/alkylation reaction of 2-MF (**1**) with different aldehydes **2a**–**d** (3.5:1 molar ratio of 2-MF to aldehyde) with para-toluenesulfonic acid (p-TosOH) as the catalyst [35].

Entry	Aldehyde	p-TosOH (mol%)	Time (h)	Temperature (°C)	Yield (%)
1	Ethanol (**2b**)	3.3	4	50	87.4
2	Ethanol (**2b**)	2.8	6	50	76.3
3	Ethanol (**2b**)	2.0	5	50	66.0
4	Propanal (**2c**)	2.6	6	50	88.4
5	Butanal (**2a**)	3.2	6	50	88.4
6	Pentanal (**2d**)	2.7	7.5	50	87.4

**Table 4:** Alkylation of 2-methylfuran with mesityl oxide.

Entry	Catalyst	Catalyst/2-MF (wt%)	Time (h)	Temperature (°C)	Yield (%)	Reference
1	p-TosOH	1.6	24	30	89	[35]
2	p-TosOH	1.4	7	60	80	[35]
3	p-TosOH	1.6	24	60	77	[35]
4	PTNT	4.5	4	20	8.2	[37]
5	Nafion212	9.1	2	60	70	[38]
6	Amberlyst36	9.1	2	60	61	[38]
7	Amberlyst15	9.1	2	60	59	[38]

**Table 5:** Synthesis of 5,5′-(furan-2-ylmethylene)bis(2-methylfuran) from 2-methylfuran and furfural.

Entry	Molar Ratio 2-MF/Furfural	Catalyst	Ratio of Catalyst/2MF	Time (h)	Temperature (°C)	Yield (%)	Reference
1	2.0	PTNT	0.0457 wt%	4	50	50	[37]
2	2.0	Nafion212	0.0457 wt%	2	65	75	[42]
3	2.0	Amberlyst36	0.0457 wt%	2	50	59	[42]
4	2.0	Amberlyst15	0.0457 wt%	2	50	60	[42]
5	2.2	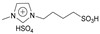	5 mol%	2	65	72	[43]
6	2.2	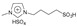	5 mol%	4	65	89	[43]
7	2.2	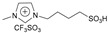	5 mol%	2	65	86	[43]
8	2.2	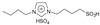	5 mol%	2	65	81	[43]
9	2.2	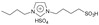	5 mol%	4	65	92	[43]
10	2.2	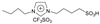	5 mol%	2	65	92	[43]
11	2.0	HC573-S423	0.0457 wt%	2	50	64	[44]
12	2.0	LF	0.0457 wt%	6	60	85	[45]
13	2.0	LF	0.0457 wt%	12	60	89	[45]
14	2.0	SHC	0.3049 wt%	5	50	83	[46]
15	2.0	Al-MSS20-450	0.0067 wt%	20	140	94	[47]
16	2.0	Formic acid	3.6552	16	55	95	[48]
17	2.0	80LS20PS350H^+^	0.0430	2	60	70	[49]
18	2.0	60LS40PS350H^+^	0.0430	2	60	88	[49]
19	2.0	LS-RES	0.0430	4	60	73	[49]
20	2.0	Amberlyst^®^70	0.0430	24	60	83	[49]
21	2.0	Amberlite^®^IR120	0.0430	24	60	84	[49]

**Table 6:** Synthesis of bis(5-methylfuran-2-yl)methane from 2-methylfuran and formaldehyde.

Entry	Molar Ratio 2-MF/Formaldehyde	Catalyst	Mass Ratio of Catalyst/2-MF	Time (h)	Temperature (°C)	Yield (%)	Reference
1	2.0	H_2_SO_4_	0.3048	3	65	74	[57]
2	2.0	Amberlyst-15	0.3048	3	65	83	[57]
3	2.0	Amberlyst IR-120	0.3048	3	65	82	[57]
4	2.0	Sn-Mont	0.3048	3	65	67	[57]
5	2.0	Zr-Mont	0.3048	3	65	60	[57]
6	2.0	[Fe_3_O_4_@SiO_2_-Pr-Py-H][2HSO_4_^2−^]	0.3048	3	65	86	[57]
7	2.0	SO_4_^2−^/SiO_2_	0.3048	3	65	74	[57]
8	2.0	SO_4_^2−^/Fe_3_O_4_@SiO_2_	0.3048	3	65	79	[57]

Reaction condition: 2-methylfuran, formaldehyde, catalyst, 65 °C, 3 h; 10 mol% of H_2_SO_4_ was used.

## Data Availability

Not applicable.
